# Attraction of *Callosobruchus chinensis* Linnaeus (Coleoptera: Bruchidae) to Volatiles Emitted by Various Leguminous Seeds

**DOI:** 10.3390/insects17080854

**Published:** 2026-08-17

**Authors:** Yu Cao, Keying Wang, Xinshuo Hu, Filippo Maggi, Paraskevi Agrafioti, Christos G. Athanassiou, Sizhe Tao, Yuqi Zhang, Fanghao Wan, Giacinto Salvatore Germinara, Shilong Jiang, Can Li

**Affiliations:** 1 College of Pharmacy, Guiyang Kangyang University, Guiyang 550081, China; yucaosuccess@126.com; 2 Guizhou Provincial Key Laboratory for Rare Animal and Economic Insect of the Mountainous Region, Guizhou Key Laboratory of Agricultural Biosecurity, Guiyang University, Guiyang 550005, China; 3 School of Pharmacy, Chemistry Interdisciplinary Project (ChIP) Research Center, Via Madonna delle Carceri, 62032 Camerino, Italy; 4 Laboratory of Entomology and Agricultural Zoology, Department of Agriculture, Crop Production and Rural Environment, School of Agricultural Sciences, University of Thessaly, 38446 Magnesia, Greeceathanassiou@uth.gr (C.G.A.); 5 State Key Laboratory for Biology of Plant Diseases and Insect Pests, Institute of Plant Protection, Chinese Academy of Agricultural Sciences, Beijing 100193, China; 6 Department of Agricultural Sciences, Food, Natural Resources and Engineering, University of Foggia, 71122 Foggia, Italy

**Keywords:** adzuki bean beetle, olfactory preference, semiochemicals, gas chromatography–mass spectrometry, electroantennography responses, stored product pests

## Abstract

*Callosobruchus chinensis* (L.) (Coleoptera: Bruchidae) is a destructive storage pest, causing serious losses to stored legume seeds. The olfactory system of these insects plays an important part in assisting them locate host plants. Olfactory responses of female *C. chinensis* adults to volatiles of disparate bean seeds (*Vigna radiata*, *Pisum sativum*, *Vigna unguiculata*, *Glycine max*, and *Phaseolus vulgaris*) were evaluated using electroantennography (EAG) and various olfactometer bioassays. In behavioral bioassays, *C. chinensis* was mostly attracted to the volatiles of *V. radiata*. We detected 37 components in the volatile profile of *V. radiata*. Among these, nonanal (11.83%), 1-hexanol (7.35%), hexanal (5.71%), tetradecane (3.83%), and 2-ethyl-1-hexanol (3.70%) were present in relatively high contents. EAG recordings indicated that the antennae of *C. chinensis* can detect these five compounds, and that adult *C. chinensis* exhibited significant positive behavioral responses to them across various concentrations. No significant differences in the olfactory preference of *C. chinensis* for nonanal were observed among different concentrations, while attraction was greatest for 1 µg/µL 1-hexanol, 25 and 10 µg/µL hexanal, 25 µg/µL tetradecane, and 1 µg/µL and 10 µg/µL 2-ethyl-1-hexanol. Based on comparisons of these compounds at their most attractive concentrations, *C. chinensis* exhibited the greatest olfactory preference for 1-hexanol. Adult female *C. chinensis* can keenly perceive volatile compounds emitted by the preferred legume *V. radiata* via their peripheral olfactory systems, and they showed positive chemotactic responses to these volatile semiochemicals. These results provide new insight into mechanisms underlying host preference in this stored-product pest. Among the compounds tested, 1-hexanol represents a potential attractant for *C. chinensis* monitoring and control.

## Simple Summary

Stored legumes are seriously threatened by insect pests, among which is the Chinese cowpea weevil, *Callosobruchus chinensis*. Understanding how this insect recognizes host seeds can support the development of environmentally friendly pest management strategies. In this study, we investigated the olfactory responses of adult female *C. chinensis* to odors released by five commonly stored legumes: mung bean, pea, cowpea, soybean, and common bean. Behavioral assays showed that mung bean produced the most attractive odor profile among the tested legumes. Chemical analysis identified 37 volatile compounds from mung bean odors, and five compounds were confirmed to be detected by the insect antennae and to influence adult behavioral responses. Among these compounds, 1-hexanol elicited the strongest attraction response. These findings provide new insights into the chemical cues involved in host selection by *C. chinensis*, and suggest that 1-hexanol could be a promising candidate for developing attractants for monitoring and managing this important stored-product pest.

## 1. Introduction

*Callosobruchus chinensis* Linnaeus (Coleoptera: Bruchidae) originated in Southeast Asia and is currently widely distributed throughout tropical and subtropical regions of the world [1,2,3]. This beetle species is highly destructive storage pest, and it poses an escalating threat to post-harvest leguminous crops like soybean, mung bean, adzuki bean, and pigeon pea [4,5]. Female *C. chinensis* adults deposit their eggs on the surface of legume seeds, after which the larvae bore into the seeds and develop internally. Larval feeding leads to pronounced quantitative losses, degradation of the nutritional quality of the stored commodity, and a substantial reduction in seed germination potential. Given its strict dependence on leguminous hosts for the completion of its life cycle, *C. chinensis* is regarded as a major and economically significant pest of stored legume crops [6,7].

The management of *C. chinensis* has traditionally relied on the use of chemical insecticides and fumigants. Although these approaches can be effective, pesticide residues may persist on treated seeds, thereby reducing their suitability for human consumption and planting. Additionally, intensified utilization of chemical insecticides has resulted in several undesirable consequences, including development of insecticide resistance and environmental contamination [8,9]. These limitations, in conjunction with growing concerns regarding environmental and food safety issues, have stimulated increasing interest in the development of alternative, more sustainable pest management strategies.

Insects inhabit highly complex and dynamic environments, and rely predominantly on their sophisticated olfactory systems for detecting and discriminating among varied chemical cues. These odor stimuli play a pivotal part in guiding insects to appropriate resources and habitats, including sites for feeding, mating, oviposition, and successful reproduction [10,11]. Prior research has established that *C. chinensis* relies on its sophisticated olfactory recognition system to precisely pinpoint host plants in natural field environments and locate stored bean resources [5,12,13]. Accordingly, behavioral manipulation represents an alternative means of pest control. A thorough understanding of pest behavior, coupled with identification of behaviorally active volatile compounds, can facilitate the development of effective lures and trapping systems for monitoring and/or control of target species, thereby offering an environmentally benign and sustainable alternative to conventional pest control methods [14,15,16].

The present study aimed to investigate the olfactory responses of adult female *C. chinensis* to seed odors from five economically important legume species. In addition, we sought to identify the volatile compounds associated with the preferred host and to evaluate the potential role in mediating host location behavior. We hypothesized that differences in seed volatile profiles contribute to the differential attractiveness of legume species and that the predominant volatile compounds are involved in the olfactory recognition of suitable hosts by female *C. chinensis*.

## 2. Methods and Materials

### 2.1. Insect Rearing

*C. chinensis* has been reared in our laboratory since 2023. *Vigna angularis* Willd. (Fabales: Fabaceae) was used as the food source to rear the insect colony in 5 L glass containers. The rearing environment was controlled at 28 ± 1 °C, with a relative humidity of 60 ± 5%, under a photoperiod of 8 h of light followed by 16 h of darkness, which aligns with the experimental conditions employed in our prior research endeavors [17,18].

### 2.2. Behavioral Responses of C. chinensis to Bean-Seed-Borne Volatiles

#### 2.2.1. Odor Sources

Five stored beans were assessed: *Vigna radiata* (Fabales: Fabaceae), *Pisum sativum* (Fabales: Fabaceae), *Glycine max* (Fabales: Fabaceae), *Phaseolus vulgaris* (Fabales: Fabaceae), and *Vigna unguiculata* (Fabales: Fabaceae), all with moisture content of 10–13%. These stored products were provided by Guiyang Grain Commodity Market (Guiyang City, Guizhou Province, China) and stored free from pesticides or pest infestation.

#### 2.2.2. Y-Tube Bioassays

Host-derived volatile compounds are important cues mediating host location and oviposition site selection in *C. chinensis*. Consequently, female adults, which are responsible for selecting suitable hosts for egg laying, represent the most relevant sex for investigating behavioral responses to seed volatiles [12,19,20]. Therefore, only female adults of *C. chinensis* were used in this study. Results for each bean seed (20.0 g) were compared with those for clean air (CA). Behavioral responses of *C. chinensis* adults (2–3 days old) were assessed utilizing a Y-tube olfactometer, following the methodology established by Cao et al. [21,22]. Airflow velocities in the individual arms of the Y-tube olfactometer were maintained at 150 mL min^−1^ using calibrated flow meters. All beetles were starved for 8 h before the test. Each female *C. chinensis* was tested individually. A single female was released at the base of the Y-tube olfactometer and observed for up to 5 min. The first choice was recorded when the beetle entered either the treatment or the control arm for at least 30 s. Individuals that failed to make a choice within 5 min were recorded as “no choice.” Forty females were tested for each stimulus, and each individual was used only once [23]. All bioassays were carried out between 09:00 and 17:00.

#### 2.2.3. Six-Arm Olfactometer Bioassays

To assess the overall attraction response of the beetles and to determine their distribution among the odor sources at a population level, group-based bioassays were performed using a six-arm olfactometer, following the methods previously recommended by Turlings et al. [24] and Cao et al. [22,25]. The six-arm olfactometer comprised a central chamber (ID = 12 cm) with six radiating arms spaced at 60° angles, each linked to a glass tube. Each glass tube was linked to a glass vessel (25.0 mL) for containing odor source (control or one of five bean seeds). Airflow was used (150 L/min) to drive odor to tested beetles. After being starved for 8 h, adults of *C. chinensis* adults (2–3 days old) were then gently transferred into the six-arm olfactometer in groups using a fine brush. Entry of *C. chinensis* adults into an olfactometer arm was a criterion for a positive response to the corresponding odor source. Each bioassay was completed within 20 min and replicated six times, with one group of 180 individuals constituting a single replicate.

### 2.3. Collection and Analysis of V. radiata Volatiles

Y-tube and six-arm olfactometer bioassays revealed that volatiles from *V. radiata* are the most attractive for *C. chinensis*. Therefore, *V. radiata* volatiles were gathered as declared by Cao et al. [22], and analyzed by using GC–MS (HP6890/5975C; Agilent Technologies, Santa Clara, CA, USA). Chromatographic separation was carried out on a ZB-5MSI fused silica capillary column (30.00 m × 250.00 μm i.d. × 0.25 μm film thickness) featuring a 5% phenyl–95% dimethylpolysiloxane stationary phase. The oven temperature program started at 40 °C, increased to 255 °C at a rate of 5 °C min^−1^, and then maintained for 2 min. The injector, transfer line, and quadrupole mass analyzer were operated at 250 °C, 280 °C, and 150 °C, respectively. The identification of target compounds was accomplished by cross-referencing their experimentally obtained mass spectra against the spectral entries stored in both the NIST 2020 and Wiley 275 reference libraries, ensuring spectral matching accuracy through dual-library validation.

### 2.4. Behavioral Responses of C. chinensis to Main V. radiata Volatiles

Given that nonanal, 1-hexanol, hexanal, tetradecane, and 2-ethyl-1-hexanol were identified as the dominant volatile compounds through GC–MS analysis, these five components were subsequently selected for further evaluation via a series of targeted bioassays, as detailed in the following sections.

### 2.5. Electroantennography

Antennal sensitivity of female *C. chinensis* to the five compounds was evaluated through EAG, as declared by Germinara et al. [26], Paventi et al. [27], and Cao et al. [21]. Briefly, the EAG setup (SYNTECH Model IDAC-2, Kirchzarten, Germany) integrated five functional modules: a high-sensitivity data acquisition unit, a precision stimulus flow regulator, a dual-mode AC/DC signal amplifier, a single-ended recording probe, and a micro-positioning manipulator. Following scalpel excision, the head of an adult beetle was positioned between two glass capillaries pre-filled with saline solution for electrode connection. We put a recording electrode in contact with the antennal club abaxial surface, and introduced a neutral electrode into the head base. Mineral oil served as a control (10 μL), and every compound was diluted in mineral oil separately to obtain varied concentrations (0.01, 0.1, 1, 10, and 100 μg/μL; 10 μL each). The EAG response of *C. chinensis* to each of the five selected compounds at increasing doses was determined utilizing the approach described by Cao et al. [18,21].

### 2.6. Behavioral Responses of C. chinensis to Five Compounds at Various Concentrations

Behavioral responses of *C. chinensis* adults to various doses of each of five compounds (nonanal, 1-hexanol, hexanal, tetradecane, and 2-ethyl-1-hexanol) were assessed using the six-arm olfactometer. Each tested compound (ranging from 0.1 to 50 μg/μL, 10 μL) or mineral oil (used as the control, 10 μL) was loaded into the odor chamber to ‌serve as an odor source [21,25]; subsequently‌, a charcoal-filtered airflow (150 mL/min) ‌delivered‌ the volatiles to *C. chinensis* to ‌assess‌ odor preference. Bioassays for each compound were repeated six times, utilizing 180 individuals for each replication.

### 2.7. Comparison Among Compounds at Their Optimal Attractive Concentrations

Nonanal, hexanol, hexanal, tetradecane, and 2-ethyl-1-hexanol were further ‌tested‌ in six-arm olfactometer bioassays (as mentioned above) at their respective optimal attractive concentrations (50, 1, 25, 25, and 10 μg/μL, respectively). A ‌steady‌ airflow of 150 mL/min carried volatiles from the six odor chambers to the test insects. Adult *C. chinensis* were ‌assayed‌ in cohorts of 180 individuals, ‌repeated six times‌.

### 2.8. Statistical Analysis

A chi-squared goodness-of-fit test (in all cases, *df* = 1) was ‌employed‌ to analyze the null hypothesis that *C. chinensis* exhibited no preference for either Y-tube arm. We checked data for normality and homoscedasticity prior to analyses. Friedman two-way ANOVA by ranks‌ was employed to compare the numbers of *C. chinensis* exhibiting odor preferences in six-arm olfactometer bioassays, followed by ‌Wilcoxon signed-rank testing‌ for pairwise comparisons where significance was detected (*p* < 0.05). Regarding to each dosage gradient of the test stimuli, the average EAG responses of female *C. chinensis* were subjected to one-way analysis of variance (ANOVA) to assess intergroup variability. Following the ANOVA, Tukey’s honest significant difference (HSD) post hoc test was implemented for pairwise comparisons between stimulus doses, with a significance threshold of *p* < 0.05 being applied to determine statistically meaningful differences. All statistical analyses were conducted using SPSS software version 22.0.

## 3. Results

### 3.1. Y-Tube Olfactometer Bioassays

**Figure 1:**
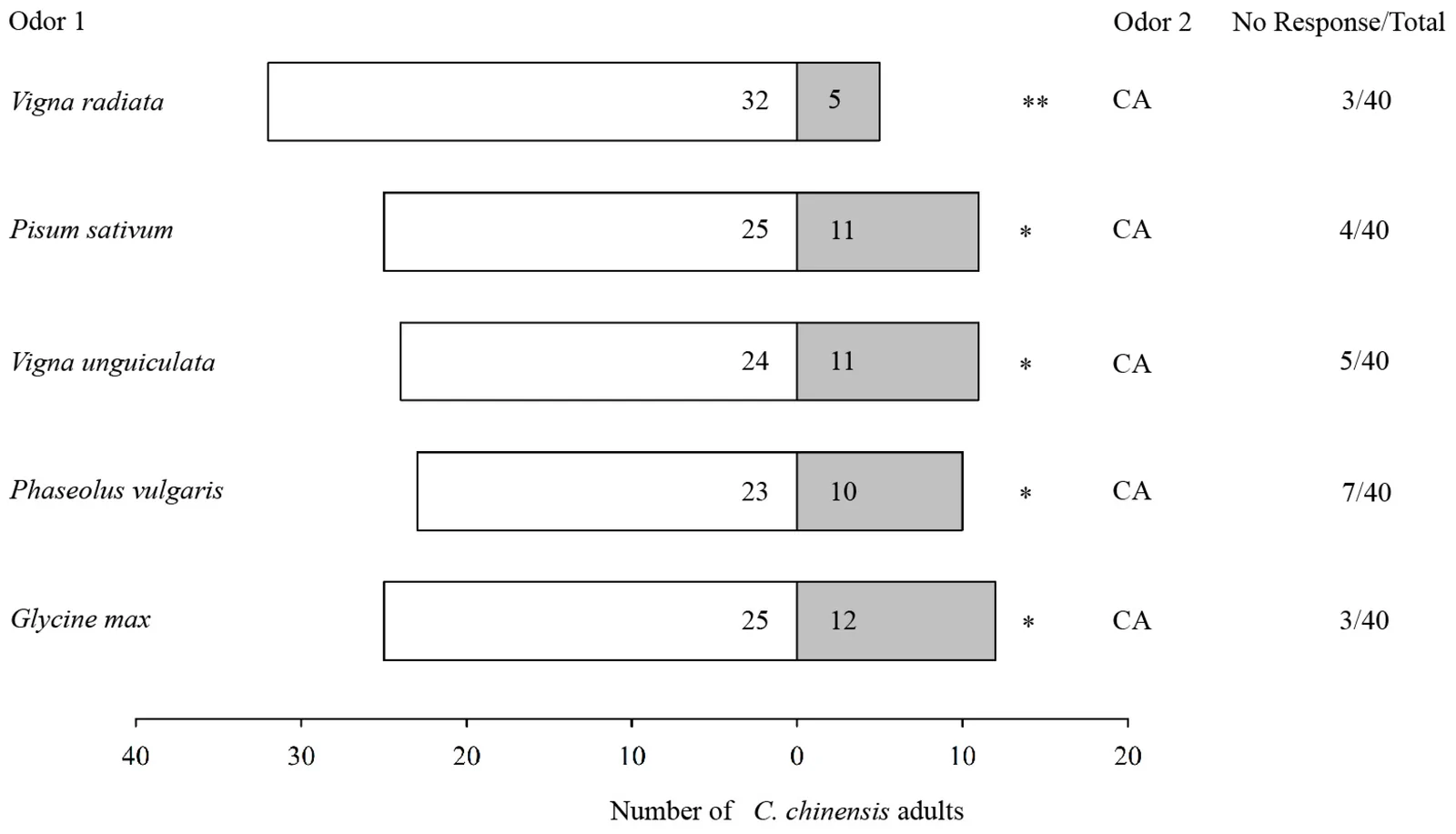
Behavioral responses of *Callosobruchus chinensis* to volatiles emitted by different bean seeds in a Y-tube olfactometer. Asterisks indicate significant (* *p* < 0.05) or highly significant (** *p* < 0.01) differences (chi-squared goodness-of-fit test).

Compared with the response to CA, *C. chinensis* displayed significant preferences for volatiles of *V. radiata* (*χ*^2^ = 19.70, *p* < 0.001), *P. sativum* (*χ*^2^ = 5.44, *p* = 0.02), *V. unguiculata* (*χ*^2^ = 4.83, *p* = 0.03), *P. vulgaris* (*χ*^2^ = 5.14, *p* = 0.02), and *G. max* (*χ*^2^ = 4.57, *p* = 0.03) (Figure 1).

### 3.2. Six-Arm Olfactometer Bioassays

**Figure 2:**
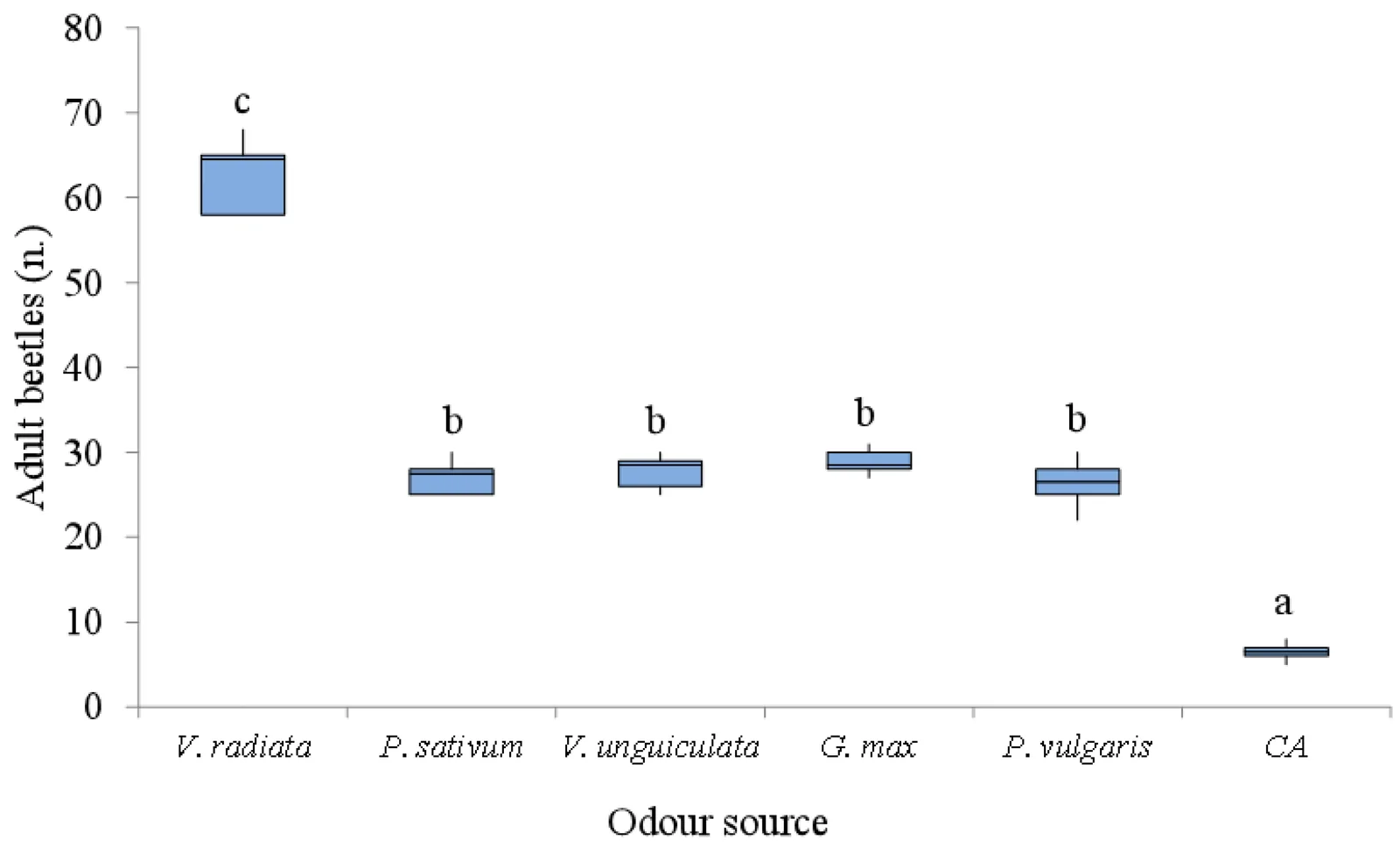
Olfactory preferences of *Callosobruchus chinensis* to odors of volatiles emitted by different bean seeds in a six-arm olfactometer. Clean air (CA) served as control. Different lowercase letters above per box plots indicate significant differences (Wilcoxon test, *p* < 0.05).

In six-arm bioassays, significantly more females were attracted to odors emitted by the five stored beans than to control air (Friedman test: *χ*^2^ = 23.52, *df* = 5, *p* < 0.001), and significantly more *C. chinensis* females were attracted to *V. radiata* than to the other beans (Wilcoxon test: *p* = 0.027). However, there were no huge disparities in the olfactory responses of *C. chinensis* to *P. sativum*, *V. unguiculata*, *P. vulgaris*, and *G. max* (Wilcoxon test: *p* > 0.05) (Figure 2).

### 3.3. Analysis of Volatiles of V. radiata

In a GC–MS analysis, 37 components were identified in the *V. radiata* volatile profile (Table 1). The top five components were nonanal (11.83%), 1-hexanol (7.35%), hexanal (5.71%), tetradecane (3.83%), and 2-ethyl-1-hexanol (3.70%). No other component occupied over 3.5% of the volatiles of *V. radiata*.

### 3.4. EAG Bioassays

**Figure 3:**
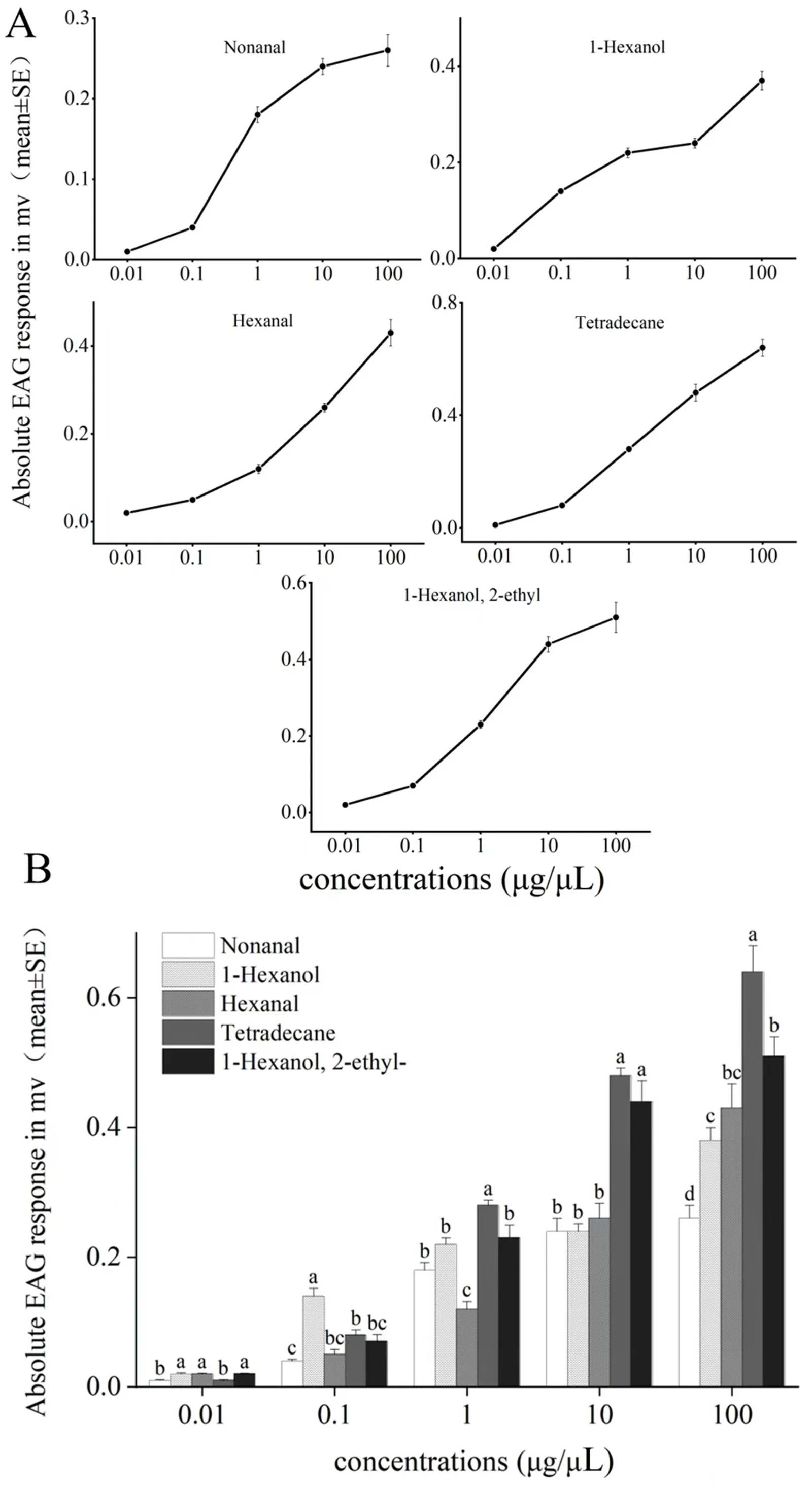
EAG responses (mean ± SE) of *Callosobruchus chinensis* to increasing doses of various compounds. Different lowercase letters above bars indicate significant differences among different compounds at the same concentration (one-way analysis of variance followed by Tukey’s HSD test, *p* < 0.05). (**A**) EAG dose–response curves of *Callosobruchus chinensis* females to increasing doses of various compounds; (**B**) EAG responses of *Callosobruchus chinensis* females to diferent doses of various compounds.

EAG responses of female *C. chinensis* to increasing doses of nonanal, 1-hexanol, hexanal, tetradecane, and 2-ethyl-1-hexanol are shown in Figure 3. In the dose range tested (beginning from 0.1 μg), all five compounds elicited typical sigmoid-shaped dose–response curves in *C. chinensis* (Figure 3A).

The responses of *C. chinensis* to the five compounds differed depending on the concentration (Figure 3B). Specifically, at 0.01 μg/μL, significantly higher EAG values were observed for 2-ethyl-1-hexanol than for other compounds; however, EAG values did not differ significantly between 1-hexanol and hexanal (*F*_4,20_ = 12.15, *p* = 0.001). Furthermore, at 0.1 μg/μL, mean EAG responses to 1-hexanol were dramatically superior to those induced by the other four compounds (*F*_4,20_ = 15.46, *p* < 0.001). At 1 μg/μL, the mean EAG response elicited by tetradecane was dramatically superior to those of the other four compounds (*F*_4,20_ = 16.26, *p* < 0.001). Among these selected test compounds, 2-ethyl-1-hexanol elicited the most robust EAG responses from female *C. chinensis* at concentrations of 10 (*F*_4,20_ = 31.73, *p* < 0.001) and 100 μg/μL (*F*_4,20_ = 20.56, *p* < 0.001), respectively. However, EAG values did not differ significantly between tetradecane and 2-ethyl-1-hexanol at 10 μg/μL (Figure 3B). 

### 3.5. Behavioral Responses of C. chinensis to the Main Compounds in V. radiata Volatiles

#### 3.5.1. Tests of Individual Compounds at Various Doses

**Figure 4:**
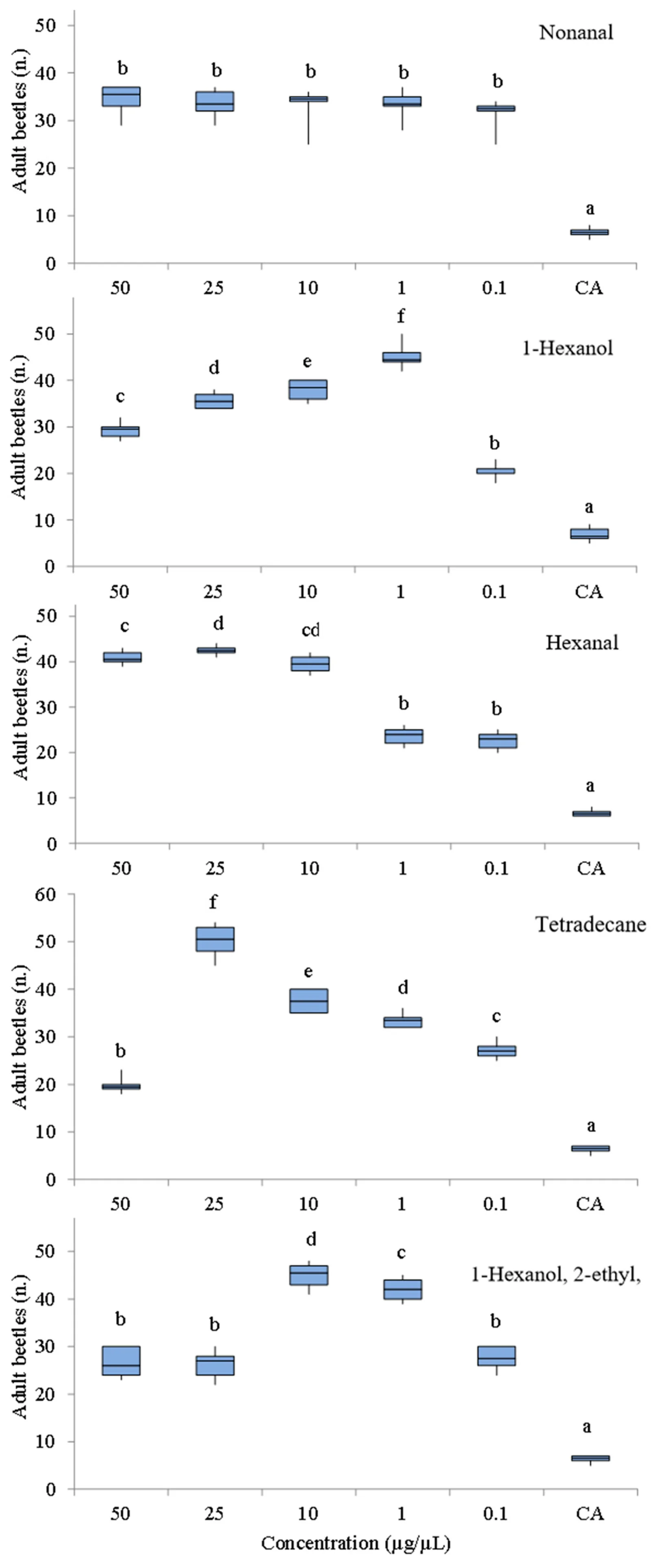
Behavioral responses of *Callosobruchus chinensis* to five volatile compounds at various doses in six-arm olfactometer assay. Mineral oil served as control. Different lowercase letters above the box plots indicate significant differences (Wilcoxon test, *p* < 0.05).

In six-arm bioassays, *C. chinensis* females showed greater attraction to all doses of nonanal (Friedman test: *χ*^2^ = 16.72, *df* = 5, *p* < 0.005, Wilcoxon tests: *p* = 0.027–0.028), 1-hexanol (Friedman test: *χ*^2^ = 30.00, *df* = 5, *p* < 0.001, Wilcoxon tests: *p* = 0.026–0.028), hexanal (Friedman test: *χ*^2^ = 27.57, *df* = 5, *p* < 0.001, Wilcoxon tests: *p* = 0.026–0.028), tetradecane (Friedman test: *χ*^2^ = 30.00, *df* = 5, *p* < 0.001, Wilcoxon tests: *p* = 0.027–0.028), and 2-ethyl-1-hexanol (Friedman test: *χ*^2^ = 27.67, *df* = 5, *p* < 0.001, Wilcoxon tests: *p* = 0.026–0.028) than to mineral oil control (Figure 4). Moreover, significant concentration-dependent differences in olfactory preferences were observed for all test compounds except nonanal. *C. chinensis* showed significantly greater olfactory preferences for 1-hexanol at 1 μg/μL, hexanal at 25 and 10 μg/μL, tetradecane at 25 μg/μL, and 2-ethyl-1-hexanol at 10 μg/μL than at other concentrations (Figure 4).

#### 3.5.2. Comparison of Compounds at Their Optimal Attractive Doses

**Figure 5:**
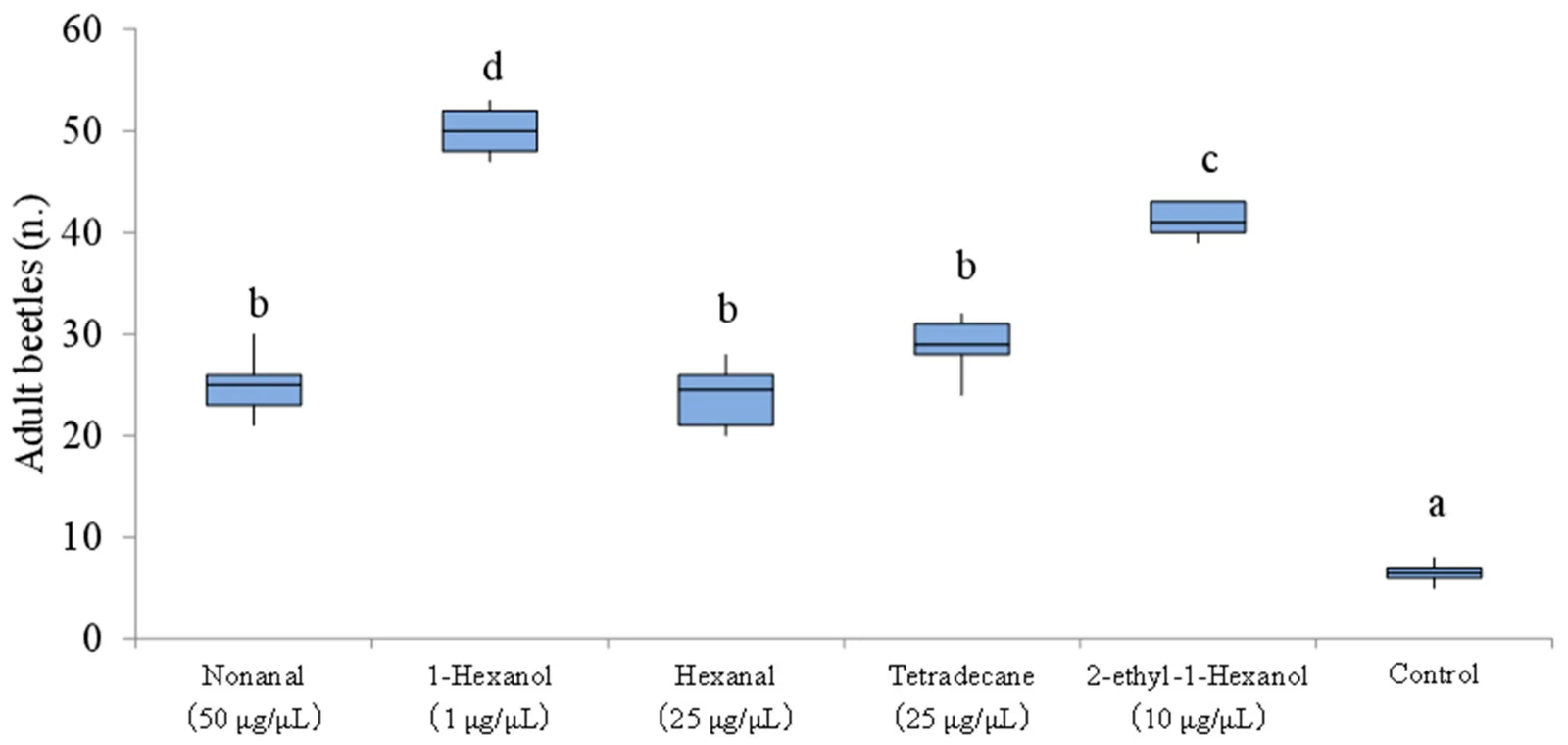
Olfactory responses of *Callosobruchus chinensis* to different compounds at their most attractive concentrations in a six-arm olfactometer. Mineral oil served as a control. Different lowercase letters above the box plots indicate significant differences (Wilcoxon test, *p* < 0.05).

Based on the results described above, nonanal, 1-hexanol, hexanal, tetradecane, and 2-ethyl-1-hexanol were further compared at their most effective concentrations in six-arm olfactometer bioassays. *C. chinensis* displayed significantly greater attraction to each of these five compounds at their optimal concentrations than to control (Friedman test: *χ*^2^ = 27.24, *df* = 5, *p* < 0.001, Wilcoxon tests: *p* = 0.024–0.028). Furthermore, *C. chinensis* exhibited a significant preference for 1-hexanol (1 μg/μL) over 2-ethyl-1-hexanol (10 μg/μL), tetradecane (25 μg/μL), hexanal (25 μg/μL), or nonanal (50 μg/μL), with no significant differences in olfactory preference between nonanal and hexanal or between nonanal and 2-ethyl-1-hexanol (Figure 5).

## 4. Discussion

Stored-product pests, e.g., *Sitophilus zeamais* Motschulsky (Coleoptera: Curculionidae), *S. oryzae* L., *S. granarius* L., and *Callosobruchus maculatus* F. (Coleoptera: Chrysomelidae), have been shown to exhibit positive olfactory responses to volatiles emitted by specific stored products [21,28,29,30,31]. In numerous insect species, volatile organic compounds emitted by host plants have been shown to play a fundamental role in localization of suitable mates, oviposition, and food source sites [32,33,34,35], as well as in behavioral avoidance of non-host substrates [36]. The systematic identification of behaviorally active chemical compounds, whether repellents or attractants, constitutes a critical scientific basis for development and implementation of effective pest monitoring and direct management strategies.

In the present study, female adults of *C. chinensis* demonstrated distinct olfactory discrimination among the volatile blends released by five legume species, exhibiting a clear preference for volatiles derived from *V. radiata*. Comparable findings for *C. chinensis* have also been reported by Fan et al. [37]. These results may partly explain why *C. chinensis* exhibits more rapid and extensive population growth on *V. radiata* compared with other legume seeds [38,39].

GC–MS analyses revealed 37 compounds in the volatile profile of *V. radiata*, among which nonanal, 1-hexanol, hexanal, tetradecane, and 2-ethyl-1-hexanol were the most abundant. EAG analyses exhibited that these five main compounds were perceived by peripheral olfactory systems of *C. chinensis* females at varied concentrations. Six-arm olfactometer bioassays established that all five compounds attracted *C. chinensis* at various concentrations, with optimal concentrations of 1 μg/μL for 1-hexanol, 25 μg/μL for hexanal and tetradecane, and 10 μg/μL for 2-ethyl-1-hexanol. There were no huge disparities in the olfactory preferences of *C. chinensis* among disparate concentrations of nonanal. Furthermore, when we compared five compounds at their most attractive concentrations, *C. chinensis* tended to prefer 1-hexanol over the other four compounds. Our results indicate that 1-hexanol exhibits the most significant potential for application as a kairomonal attractant targeting *C. chinensis*.

In stored-product pest species, responses to volatile compounds are often species-dependent. *Sitophilus granarius* (L.) (Coleoptera: Curculionidae) exhibits significant olfactory attraction to volatiles emitted by specific stored grain species [21]. *Araecerus fasciculatus* De Geer (Coleoptera: Anthribidae) is attracted to *β*-elemene, as identified from theb volatile profile of the Chinese medicinal plant *Ophiopogon japonicus* (Asparagales: Asparagaceae) [40]. 3-n-Butylphthalide, the most abundant compound in the volatile profile of another Chinese medicinal plant, *Angelica sinensis* Oliv. Diels (Apiales: Apiaceae), is highly attractive to *Stegobium paniceum* (L.) (Coleoptera: Anobiidae) [18]. Nonanal is not the compound most attractive to *C. chinensis*, but it is attractive to *S. oryzae* and *Oryzaephilus surinamensis* (L.) (Coleoptera: Silvanidae) [41,42]. Hexanal is commonly used as an attractant for important secondary pests of processed cereals, like *O. surinamensis* and *Oryzaephilus mercator* Fauvel (Coleoptera: Silvanidae) [43]; however, it exhibits a strong repellent effect on *S. granarius* adults [30]. Additionally, several studies have emphasized the critical role of volatile compound ratios and concentrations in the host-finding behavior of phytophagous insects [44,45,46]. In light of our findings, it is critical to systematically assess the efficacy of varying dosages and combinatorial formulations of the kairomones identified in this study.

Only a small subset of the volatile compounds released by plants serve as key chemical cues that guide phytophagous insects in detecting and locating their hosts [47,48]. Olfactory responses of *C. chinensis* to only the five main compounds were measured individually in the present study. Further studies should screen for more bioactive compounds (individual compounds or blends at various concentrations) that attract *C. chinensis*. In-depth studies on the interactions between olfactory system of *C. chinensis* and volatile compounds are also essential to deeply elucidate the molecular mechanism of olfactory recognition in species, and to identify novel RNAi targets for controlling this pest in the future [5,13,49].

## Figures and Tables

**Table 1:** Volatile organic compounds of *Vigna radiata* volatiles.

Number	Compound	Retention Time	RI	Molecular Formula	Relative Content (%)
1	Ethanol	1.635	753	C_2_H_6_O	0.60
2	Acetone	1.713	755	C_3_H_6_O	1.06
3	Acetic acid	2.105	763	C_2_H_4_O_2_	1.11
4	Furan, 2-ethyl-	3.086	785	C_5_H_10_O_2_	0.71
5	1-Butanol, 3-methyl-	3.621	797	C_5_H_12_O	0.96
6	Toluene	4.249	810	C_7_H_8_	1.44
7	1-Pentanol	4.279	811	C_5_H_12_O	0.83
8	2,3-Butanediol	4.609	818	C_4_H_10_O_2_	0.77
9	Octane	5.008	827	C_8_H_18_	2.40
10	Hexanal	5.021	827	C_6_H_12_O	5.71
11	3-Furaldehyde	5.440	837	C_10_H_14_	1.42
12	1-Hexanol	7.206	876	C_6_H_14_O	7.35
13	Heptanal	8.380	901	C_7_H_14_O	0.65
14	Oxime-, methoxy-phenyl-	8.655	907	C_8_H_9_NO_2_	3.31
15	*α*-Pinene	9.592	928	C_10_H_16_	0.69
16	Furan, 2-pentyl-	12.215	986	C_9_H_14_O	1.14
17	Limonene	13.816	1021	C_10_H_16_	2.01
18	1-Hexanol, 2-ethyl-	13.965	1024	C_8_H_18_O	3.71
19	Benzyl alcohol	14.178	1029	C_6_H_10_O_2_	2.13
20	1-Octanol	15.957	1068	C_8_H_18_O	2.29
21	Nonanal	17.474	1101	C_9_H_18_O	11.83
22	Phenylethyl Alcohol	17.880	1111	C_8_H_10_O	1.88
23	Isophorone	18.122	1116	C_9_H_14_O	1.39
24	Undecane,3-methyl-	20.598	1170	C_12_H_26_	0.68
25	1-Nonanol	20.717	1172	C_9_H_20_O	0.54
26	Naphthalene	20.945	1178	C_10_H_8_	2.76
27	Dodecane	21.970	1200	C_12_H_26_	2.11
28	Decanal	22.237	1206	C_10_H_20_O	1.54
29	Tridecane	26.492	1300	C_13_H_28_	1.03
30	Tetradecane	30.813	1397	C_14_H_30_	3.83
31	Longifolene	30.869	1399	C_15_H_24_	2.62
32	Tetradecane, 3-methyl-	33.708	1466	C_15_H_32_	0.90
33	Pentadecane	34.919	1494	C_15_H_32_	1.19
34	Pentadecane, 4-methyl-	37.173	1551	C_16_H_34_	0.75
35	Pentadecane, 3-methyl-	37.660	1564	C_16_H_34_	1.51
36	Hexadecane	38.773	1592	C_16_H_34_	0.82
37	Heptadecane	42.151	1698	C_18_H_38_	1.49

## Data Availability

The original contributions presented in this study are included in the article. Further inquiries can be directed to the corresponding authors.
